# Left ventricular geometry during unloading and the end-systolic pressure volume relationship: Measurement with a modified real-time MRI-based method in normal sheep

**DOI:** 10.1371/journal.pone.0234896

**Published:** 2020-06-22

**Authors:** Duc M. Giao, Yan Wang, Renan Rojas, Kiyoaki Takaba, Anusha Badathala, Kimberly A. Spaulding, Gilbert Soon, Yue Zhang, Vicky Y. Wang, Henrik Haraldsson, Jing Liu, David Saloner, Julius M. Guccione, Liang Ge, Arthur W. Wallace, Mark B. Ratcliffe

**Affiliations:** 1 Veterans Affairs Medical Center, San Francisco, California, United States of America; 2 Department of Bioengineering, University of California, San Francisco, CA, United States of America; 3 Department of Surgery, University of California, San Francisco, CA, United States of America; 4 Department of Radiology, University of California, San Francisco, CA, United States of America; 5 Department of Anesthesia, University of California, San Francisco, CA, United States of America; University of Alberta, CANADA

## Abstract

The left ventricular (LV) end-systolic (ES) pressure volume relationship (ESPVR) is the cornerstone of systolic LV function analysis. We describe a 2D real-time (RT) MRI-based method (RTPVR) with separate software tools for 1) semi-automatic level set-based shape prior method (LSSPM) of the LV, 2) generation of synchronized pressure area loops and 3) calculation of the ESPVR. We used the RTPVR method to measure ventricular geometry, ES pressure area relationship (ESPAR) and ESPVR during vena cava occlusion (VCO) in normal sheep. 14 adult sheep were anesthetized and underwent measurement of LV systolic function. Ten of the 14 sheep underwent RTMRI and eight of the 14 underwent measurement with conductance catheter; 4 had both RTMRI and conductance measurements. 2D cross sectional RTMRI were performed at apex, mid-ventricle and base levels during separate VCOs. The Dice similarity coefficient was used to compare LSSPM and manual image segmentation and thus determine LSSPM accuracy. LV cross-sectional area, major and minor axis length, axis ratio, major axis orientation angle and ESPAR were measured at each LV level. ESPVR was calculated with a trapezoidal rule. The Dice similarity coefficient between LSSPM and manual segmentation by two readers was 87.31±2.51% and 88.13±3.43%. All cross sections became more elliptical during VCO. The major axis orientation shifted during VCO but remained in the septo-lateral direction. LV chamber obliteration at the apical level occurred during VCO in 7 of 10 sheep that underwent RTMRI. ESPAR was non-linear at all levels. Finally, ESPVR was non-linear because of apical collapse. ESPVR measured by conductance catheter (E_ES,Index_ = 2.23±0.66 mmHg/ml/m^2^) and RT (E_ES,Index_ = 2.31±0.31 mmHg/ml/m^2^) was not significantly different. LSSPM segmentation of 2D RT MRI images is accurate and allows calculation of LV geometry, ESPAR and ESPVR during VCO. In the future, RTPVR will facilitate determination of regional systolic material parameters underlying ESPVR.

## Introduction

The left ventricular (LV) end-systolic pressure (ES) volume relationship (ESPVR) is the cornerstone of LV systolic function analysis [1, 2]. For instance, the slope of the ESPVR (end-systolic elastance (E_ES_)), which is usually measured during a transient reduction in preload (vena cava occlusion (VCO)) [3], is widely used in basic and clinical research as a load independent measure of global LV systolic function [4].

However, there are several gaps in our knowledge of ESPVR. First, at the myocyte level, it is generally accepted that the active force myocyte/sarcomere length relationship determines the ESPVR [5, 6] but little is known about how underlying systolic material properties relate to ESPVR. Second, LV systolic function is regionally heterogeneous in both the normal LV [7] and in patients with ischemic [8] and valvular heart disease [9, 10] but little is known about how regional systolic function contributes to the ESPVR. Imaging of the LV during VCO is a necessary prerequisite for investigation in both of these areas.

Conventional ESPVR methods including sonomicrometry [11] and the conductance catheter [12] provide limited LV geometry data. The 3D location of sonomicrometry transducers can be determined with the array localization method [13], however, the number of transducers is limited, wall thickness is usually measured at a single point [7] and calculation of LV geometry must assume an ideal LV shape [10]. Dahl et al measured both ESPVR with a conductance catheter and strain and strain rate with 2D echocardiography during VCO in normal pigs but LV geometry was not reported [14]. Segmental LV volume data can be obtained from a conductance catheter [15] but this provides only indirect information about segmental LV shape.

Although conventional cine cardiac magnetic resonance imaging (CMRI) has excellent spatial resolution, acquisition times were not previously adequate for imaging during preload reduction. However, Zhang et al recently described a real-time CMRI (RTMRI) technique based on a radial FLASH sequence with nonlinear inverse reconstruction that has image acquisition times as short as 20 to 30 ms [16] and Witschey et al used the RTMRI method to acquire 2D images of the LV during VCO in normal sheep [17]. LV pressure area loops were created and the ES and ED pressure area relationships (ESPAR; EDPAR) were measured at a range of levels between the LV apex and base [18]. Nevertheless, the alterations in LV geometry during VCO have not been studied previously.

Both active contour methods [19–21] and deep learning methods [22–24] have been successfully used for automatic segmentation of the LV cavity and wall. Deep learning methods require training sets that are large and rich while active contour methods which are usually based on image intensity and shape do not require training sets. Given that training sets of the LV cavity during VCO would be difficult to obtain, in the current study we chose to employ the active contour-type level set shape prior method (LSSPM) described by Wang et al. [19] for automatic segmentation of the LV during VCO. LSSPM has been validated with an MRXCAT phantom [25] and 17 human MRI studies with over 5000 2D images and achieved the highest Dice value [26] compared with three state-of-the-art published methods. LSSPM is able to segment objects with a changing evolving shape and is therefore well suited for segmentation of the LV during VCO.

We describe a 2D RTMRI-based method (RTPVR) that includes separate software tools for 1) LSSPM segmentation of the LV cavity, 2) generation of synchronized pressure area loops and 3) calculation of the ESPVR. We then use the method to measure ventricular geometry during VCO and corresponding ESPAR and ESPVR in normal sheep.

## Methods

### Overview

Software tools for 1) automatic level set-based shape prior method (LSSPM) segmentation of the LV (Matlab R2016b, Mathworks, Natick, MA), 2) generation of synchronized pressure area loops and 3) calculation of the ESPVR were created (C#, Visual Studio 2017, Microsoft, Redmond, WA with Matlab.net compiler). We first validated the LSSPM method of LV cavity segmentation by comparing manual segmentation performed by two image readers with LSSPM output. The Dice similarity coefficient, which determines the similarity of two image objects as the ratio of twice the object intersection over the union of the image objects, is commonly used to determine the accuracy of image segmentation methods [26]. Accordingly, intra- and inter-observer variabilities and the Dice coefficient were calculated to determine the accuracy of the LSSPM method.

**Fig 1:**
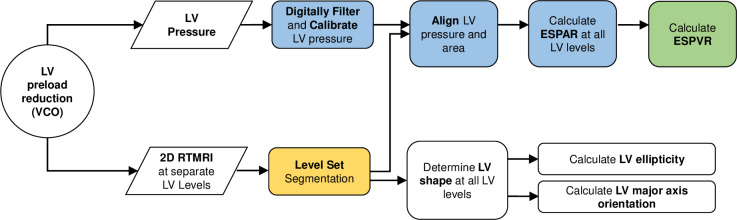
A flowchart illustrating the overall methodology designed to derive subject-specific ESPVR from RTMRI and LV pressure catheter measurement.

A flowchart illustrating the RTPVR method is shown in **Fig 1**. Briefly, separate 2D short axis RTMRI was performed at apex, mid-ventricle and base LV levels during separate VCOs. LSSPM calculated LV short axis geometry was used to calculate major and minor axes, axis ratio and axis orientation. LV pressure and area are then synchronized and ESPAR loops are generated. Mixed model regression was used to determine the effect of LV level on major and minor axes, axis orientation and ESPAR slope and intercept. ESPVR is then calculated using the trapezoidal rule method [27].

### Experimental animals

Studies were approved by the San Francisco VA Institutional Animal Care and Use Committee (IACUC), in compliance with the “Guide for the Care and Use of Laboratory Animals” prepared by the Institute of Laboratory Animal Resources, National Research Council.

Sheep were acquired from a local ranch (Pozzi Ranch, Sebastapol, CA). A Q-fever titer was acquired prior to animal purchase and then twice during a three week quarantine at the animal facility. Only sheep with three negative titers were used.

### Cardiac MRI (CMRI)

Fourteen healthy, adult sheep were sedated with ketamine (20mg/kg intravenous), anesthetized (Isoflurane 2.2% inhaled) and mechanically ventilated. The tip of a pressure catheter (SPC-350; Millar, Houston, TX) was immersed in water at 38°C for 12 hours prior to calibration and positioning in the LV and an 8 Fr balloon catheter was positioned in the inferior vena cava via femoral vessels using fluoroscopic guidance. Ferumoxytol (0.125 ml/kg IV over 1 hour; AMAG Pharmaceuticals, Waltham, MA) was given 1 hour prior to MRI [28]. Of note, Ferumoxytol provides superior blood pool contrast that does not fade during the repeated VCO necessary for the RT ESPVR measurements [29]. Metoprolol (5 mg) and atropine (1 mg) were also given intravenously immediately prior to MRI. Isoflurane was maintained at 2.2%; end-tidal CO_2_ was kept between 25 and 45 mm Hg; an infusion of neosynephrine was titrated to keep peak LV pressure at 90+5 mm Hg during cardiac MRI.

MRI imaging was performed on a 3T MRI scanner (Skyra; Siemens, Erlangen, Germany). Six cine long axis MR images that were 30^o^ apart were used for LV volume (LVV(CineMRI)) calculation [30].

Ten of the 14 sheep underwent measurement of LV systolic function with RTMRI methods. 2D short axis RTMRI was performed 25 (Apex), 50 (Mid) and 75% (Base) of the distance from the apex to the base of the LV during separate VCOs with ventilation temporarily suspended. The MR imaging parameters are summarized as follows: 2D multislice retrospectively-gated cine balanced steady-state free-precession acquisition with the following imaging parameters, TE = 1.34 ms, TR = 59.2 ms, acquisition matrix = 128 x 54, FOV = 178 x 260, slice thickness = 8 mm, pixel spacing = 2.0313 x 2.0313 mm, temporal resolution = 59.2 msec.

**Fig 2:**
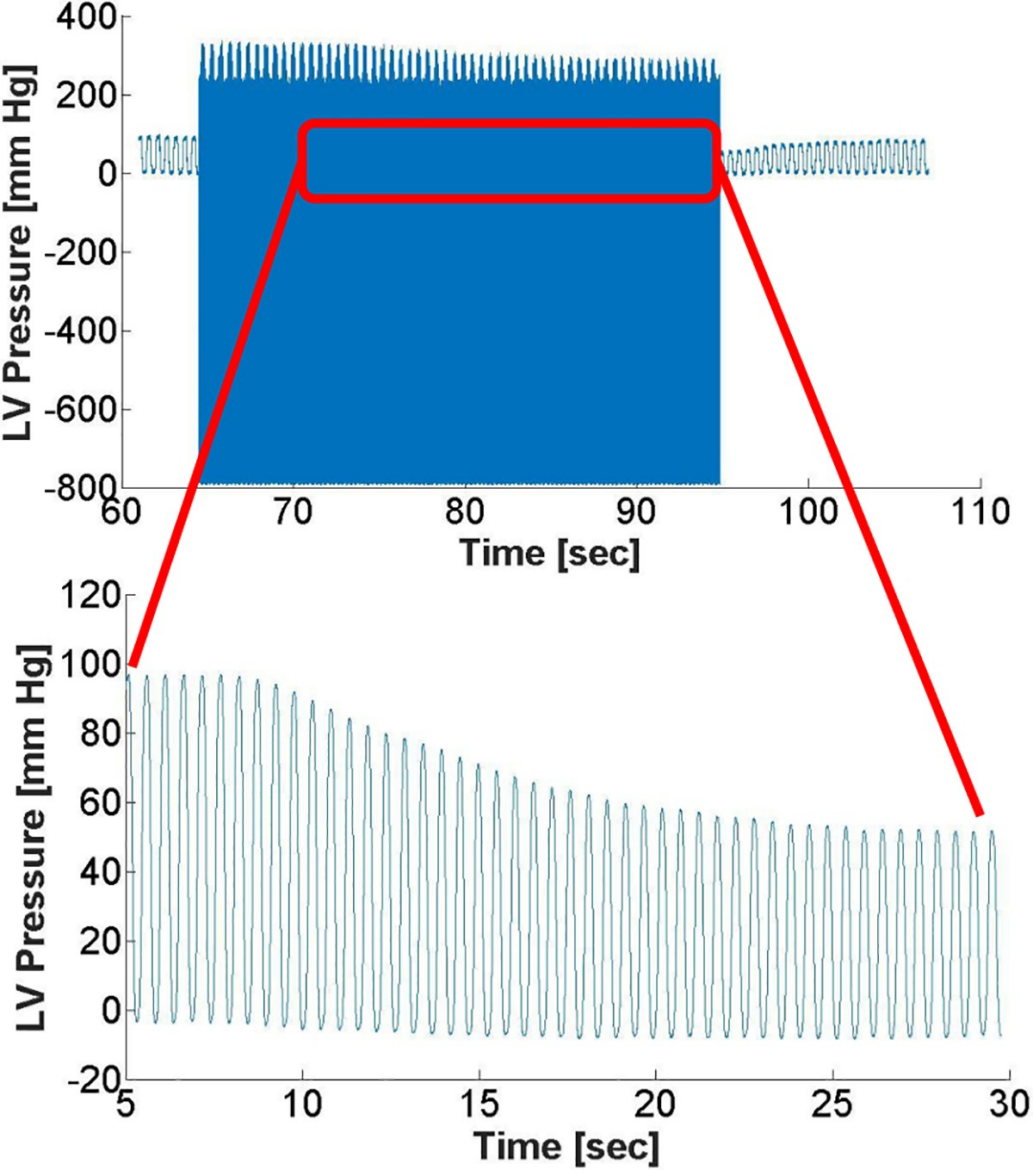
Raw pressure data with noise prior to digital filtering and calibration (top) with the insert showing filtered and calibrated LV pressure during the selected VCO range.

LV pressure (LVP) acquisition (12 bit, 5K samples/ sec, ACQ16 and Ponemah 5.2, DSI, St. Paul, MN) started approximately 5 seconds before the start of the MRI acquisition as shown in **Fig 2**. LV pressure was filtered with an in-line analog filter (BNC Low Pass Filter, Crystek, Fort Myers, FL) during acquisition and post-processed with a digital constrained least Pth-norm IIR filter (Matlab). LVP during VCO was calibrated using pressure acquired immediately prior to MRI acquisition as shown in **Fig 2B**.

### Conductance catheter measurements

As the current standard of ESPVR measurement is the conductance catheter, eight of the 14 sheep also underwent measurement of LV systolic function with a conductance catheter and of those 4 had both RT and conductance measurements. Following cine and RTMRI, the LV pressure catheter was removed and a 5F twelve electrode conductance catheter (Ventri-Cath 510; Millar) introduced via arterial introducer and 6F guide catheter (CGC7100MP1, Merit Medical, Jordan, UT). The conductance catheter was positioned along the LV long axis with fluoroscopic guidance. Conductance catheter measurements were obtained during VCO with ventilation temporarily suspended. Conductance catheter output was processed (MPVS Ultra; Millar) and digitized (Ponemah).

### Validation of semi-automatic level set-based shape prior method (LSSPM) segmentation

**Fig 3:**
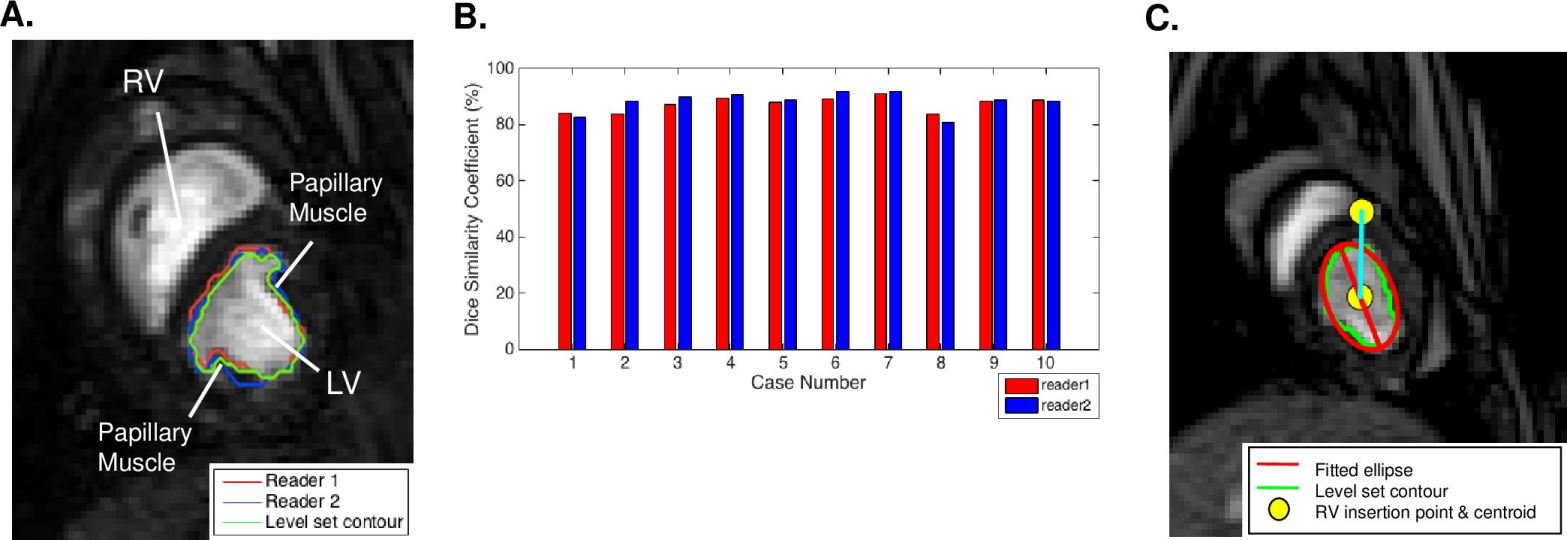
**(A)** Representative midventricular short-axis image of the LV showing level set and manual contours. Green = automatic contouring, Red = manual contour reader 1, Blue = manual contour reader 2. **(B)** Dice similarity coefficient for all 10 cases. (**C**) Representative midventricular short-axis image of the LV overlaid with automatic contours and fitted ellipse for quantifying major/minor axis length and major axis orientation angle as surrogate measurement of LV shape change. Red = fitted ellipse. Green = automatic contouring. Yellow circle = RV insertion point and centroid of ellipse.

LSSPM was designed for cardiac segmentation and functional measurements by Wang et al. [19]. In our case, manual segmentation was performed in a blinded manner by 2 readers who contoured 200 slices from all 10 subjects (20 slices for each case) as shown in **Fig 3A**. A second set of manual contours was drawn by each of the readers in 3 of the cases allowing the calculation of both inter- and intra-observer variability. The Dice similarity coefficient, commonly used to determine the accuracy of image segmentation methods, was used to determine the accuracy of the LSSPM contours (**Fig 3B**) [26].

### Image segmentation

The LSSPM was used to segment the 2D RTMRI images [31]. Specifically, the first frame of each imaging series was manually contoured while subsequent frames were processed with the LSSPM [31].

LSSPM generated contours were used to calculated LV cross-sectional area. In addition, an ellipse was fit to the LSSPM generated contours and major and minor axes, axis ratio, and major axis orientation angle were calculated. Note that the orientation was relative to the anterior right ventricular endocardial insertion and the LV septum was in the positive direction **(Fig 3C)**.

**Fig 4:**
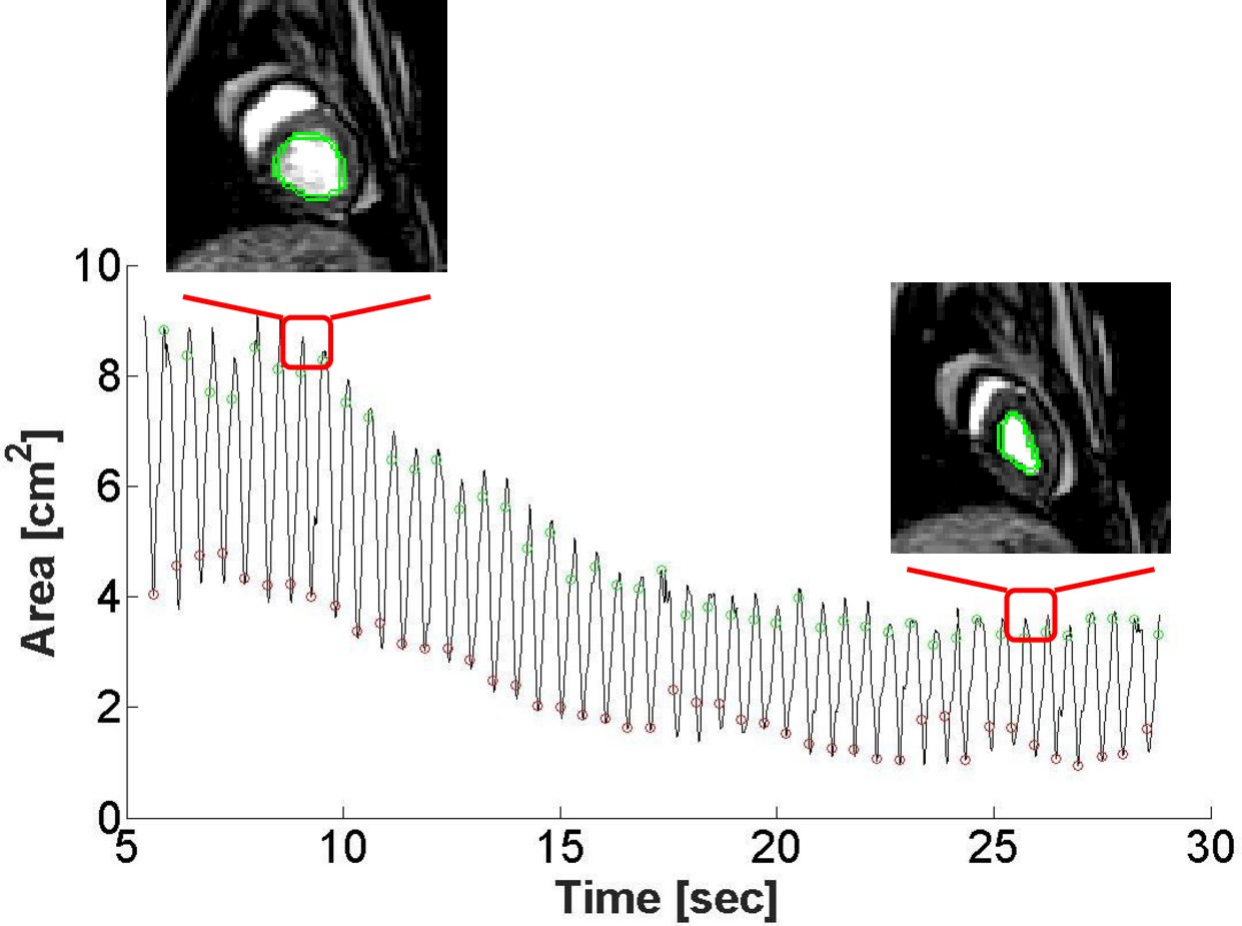
LV Area plot calculated by the level set algorithm with representative midventricular short-axis images of the LV before VCO and during VCO starting at t = 10 sec. Green makers = end-diastole (ED), Red markers = end-systole (ES).

Obliteration of the LV cavity was defined as LV area ≤ 0.25 cm^2^. In instances where the LV cavity collapsed, axis and angle data were excluded. Representative LV shape and area changes during VCO are shown in **Fig 4**.

### ESPAR calculation

LV area was initially aligned to the region of noise on the unfiltered LVP trace. The time derivative of the LVP (d LVP/dt) was calculated and 0.25 peak d LVP/dt was aligned with peak LV area. The alignment method was chosen to obtain optimal LV pressure area loops (**S1 Fig**). The ESPAR was calculated using a shooting point iterative regression approach previously described by Kono et al for ESPVR determination from conductance catheter data [32].

### ESPVR calculation

Real time LV volume at ES (*LVV*(*RTMRI*)_*ES*_) was determined with the trapezoidal rule [27] as follows:

$$LVV{\left(RTMRI\right)}_{ES,LVP}=\sum_{i=1}^{5}\frac{1}{2}\left(\left(LVA_{ES,i}+LVA_{ES,i+1}\right)\left(LVDist_{i+1}-LVDist_{i}\right)\right)+LVV{\left(RT\right)}_{ES,LVP=90}$$ (1)

where *LVA*_*ES*,*i*_ is LV cross-sectional area with i ranging from 1 (LV apex) to 5 (LV valve plane), *LVDist*_*i*_ is the distance of the real time MRI short-axis slices from the LV apex. Note that *LVDist*_5=*ValvePlane*_ is assumed to move toward the apex in line with sonomicrometry data obtained in conscious dogs during VCO [33]. *LVV*_*ES*,*0*_ is the baseline ES volume calculated from step 1 and is assumed to be constant during the VCO. Subsequent points on the ESPVR are generated using *LVA*(*P*)_*i*_ at LVP range between 50 and 90 mmHg with a 5 mm Hg step change.

### Conductance catheter analysis

At least 10 beats during VCO were selected and analyzed. Similar to RTMRI, conductance catheter ES was identified as the point of maximal elastance. [32]. LVV(CineMRI) was used to calculate the gain (α) and parallel conductance offset of the conductance catheter measurements. Specifically,

$$LVV(Cond,Corrected)(t)=\alpha \left(LVV(Cond)(t)-LVV{\left(Cond\right)}_{ES}\right)+LVV{\left(CineMRI\right)}_{ES}$$ (2)

where:

$$\alpha =\left(LVV{\left(CineMRI\right)}_{ED}-LVV{\left(CineMRI\right)}_{ES}\right)/\left(LVV{\left(Cond\right)}_{ED}-LVV{\left(Cond\right)}_{ES}\right)$$ (3)

The method of conductance catheter calibration with MRI has been described previously [34].

RTMRI and conductance catheter-based ESPVR calculation was performed with custom code written in C# (Visual Studio 2017; Microsoft, Redmond, WA) using Matlab routines (.NET assembly using Matlab Compiler, Mathworks; https://github.com/mratcliffe/PVAnalysis, available on request).

### Statistical analysis

All values are expressed as mean ± standard error of the mean. The significance level was set at p<0.05.

Multivariate mixed effects analyses (Proc Mixed, SAS version 9.2, SAS Institute Inc., Cary, NC) were used to determine changes in variables including cardiac cycle length, ED-ES interval, peak systolic pressure, LV area, major and minor axis and axis orientation between and during VCO. Individual sheep were included as a random effect [35].

Mixed effects analysis of RT ESPVR included both linear and quadratic terms. RT and conductance catheter calculations of E_ES_ were compared with unpaired t-test.

## Results

### Semi-automatic level set-based shape prior method

LSSPM segmentation was automatic in 7 of 10 sheep data sets and required minor parameter modifications in the remaining 3 of 10 data sets. The average number of images per beat was 10.76 and the computation time for automatic segmentation was less than 10 seconds per slice. The intra-observer variability from the two readers were 0.54% and 0.88%. The Dice similarity coefficient between level set segmentation and manual segmentation by two readers was determined to be 87.31%±2.51% and 88.13%±3.43% respectively thus demonstrating high LSSPM accuracy as shown in **Fig 3B**. A representative LV area vs time plot is shown in **Fig 4**.

### Cardiac cycle timing and pressure

Cycle length, ED-ES interval and LV pressure at baseline are seen in **Table 1**. The average baseline sheep heart rate is 94.19 bpm. The ED-ES interval was significantly shorter at the mid ventricle level (0.246+0.005 sec) than at the apical level (0.261+0.005 sec) but the cardiac cycle length was not different between LV levels. At baseline, the LVP was significantly lower at the apical level (88.6 ± 1.60 mm Hg) than both the mid-ventricle and basal levels (94.8 ± 1.34 and 95.1 ± 1.40 mm Hg respectively).

**Table 1:** Baseline cardiac MRI measurements.

	All VCOs (n = 30)	Apex (n = 10)	Mid (n = 10)	Base (n = 10)	Significance*
Cycle Length [sec]	0.637+0.007	0.631 + 0.008	0.640 + 0.012	0.641+ 0.014	NS
ED-ES Interval [sec]	0.255+0.003	0.261 + 0.005	0.246 + 0.005	0.257 + 0.005	1, 3
LV Pressure at ES [mm Hg]	92.9+0.9	88.6 + 1.6	95.1 + 1.4	94.8 + 1.3	2, 3
LV Area at ES [cm^2^]	5.43+0.25	2.53 + 0.156	4.55 + 0.181	9.15 + 0.323	1, 2, 3
LV Major Axis at ES [cm]	2.77+0.06	1.99 + 0.0601	2.637 + 0.0573	3.66 + 0.0627	1, 2, 3
LV Minor Axis at ES [cm]	2.37+0.06	1.63 + 0.0622	2.27 + 0.0456	3.20 + 0.0576	1, 2, 3
LV Axis Ratio at ES	1.19+0.01	1.26 + 0.0307	1.16 + 0.0150	1.15 + 0.0129	2, 3
Major Axis Orientation Angle at ES [^o^]	31.7+3.7	13.7 + 7.46	51.2 + 5.67	29.9 + 4.74	1, 2, 3

Baseline cardiac cycle length, end-diastole to end-systolic (ED-ES) time interval, LV pressure and area, major and minor axis, axis ratio and axis angle at ES for all VCO data and at three LV levels.

*NS: not significant, 1: Base vs Mid (p<0.05), 2: Base vs Apex (p<0.05), 3: Mid vs Apex (p<0.05).

Changes in cycle length, ED-ES interval and LV pressure during VCO are seen in **Table 2**. There was statistically significant shortening of both the ED-ES interval (-0.0038+0.0005 sec/ beat) and cardiac cycle length (-0.0024+0.0004 sec/ beat) during VCO. For reference, this is a 4.8 msec decrease in cycle length over a 20 beat VCO. The rate of change of LVP during VCO was not different between LV levels.

**Table 2:** VCO cardiac MRI measurements.

	All VCOs (n = 30)	Apex (n = 10)	Mid (n = 10)	Base (n = 10)	Significance*
Cycle Length [sec/ beat]	-0.0038+0.0005	-0.0027+0.0008	-0.0043+0.0011	-0.0042+0.0006	1
ED-ES Interval [sec/ beat]	-0.0024+0.0004	-0.0035+0.0009	-0.0018+0.0008	-0.0020+0.0004	1
LV Pressure at ES [mm Hg/ beat]	-2.26+0.11	-2.32+0.19	-2.23+0.21	-2.22+0.18	1
LV Area at ES [cm^2^/ beat]	-0.13+0.02	-0.08+0.02	-0.11+0.01	-0.21+0.04	1,2
LV Major Axis at ES [cm/ beat]	-0.038+0.003	-0.033+0.008	-0.040+0.004	-0.043+0.0058	1
LV Minor Axis at ES [cm/ beat]	-0.042+0.004	-0.033+0.009	-0.041+0.004	-0.050+0.008	1
LV Axis Ratio at ES	0.0080+0.0017	0.0084+0.0041	0.0072+0.0020	0.0085+0.0026	1
Major Axis Orientation Angle at ES [^o^/ beat]	-0.19+0.28	-1.04+0.46	-0.34+0.54	0.81+0.27	1,2

Slope of cycle length, end-diastole to end-systolic (ED-ES) time interval, LV pressure and area, and major and minor axis, axis ration and axis angle at ES vs VCO beat for all VCO data and at three LV levels.

*1: All VCO variables vs beat (p<0.05), 2: Base vs Apex (p<0.05).

### LV geometry

LV cross-sectional area, major and minor axes, axis ratio, and major axis orientation are shown in **Table 1**. As expected, LV area, major axis, and minor axis lengths increased from the apical to the basal segment. Conversely, axis ratios decreased from the apical to the basal segment, confirming that the LV is more spherical at the basal segment and more elliptical at the apical segment. Major axis orientation angle was greatest at the mid-ventricle level with an average angle of 51.2° while the base and apex levels had average angles of 29.9° and 13.7° respectively.

**Fig 5:**
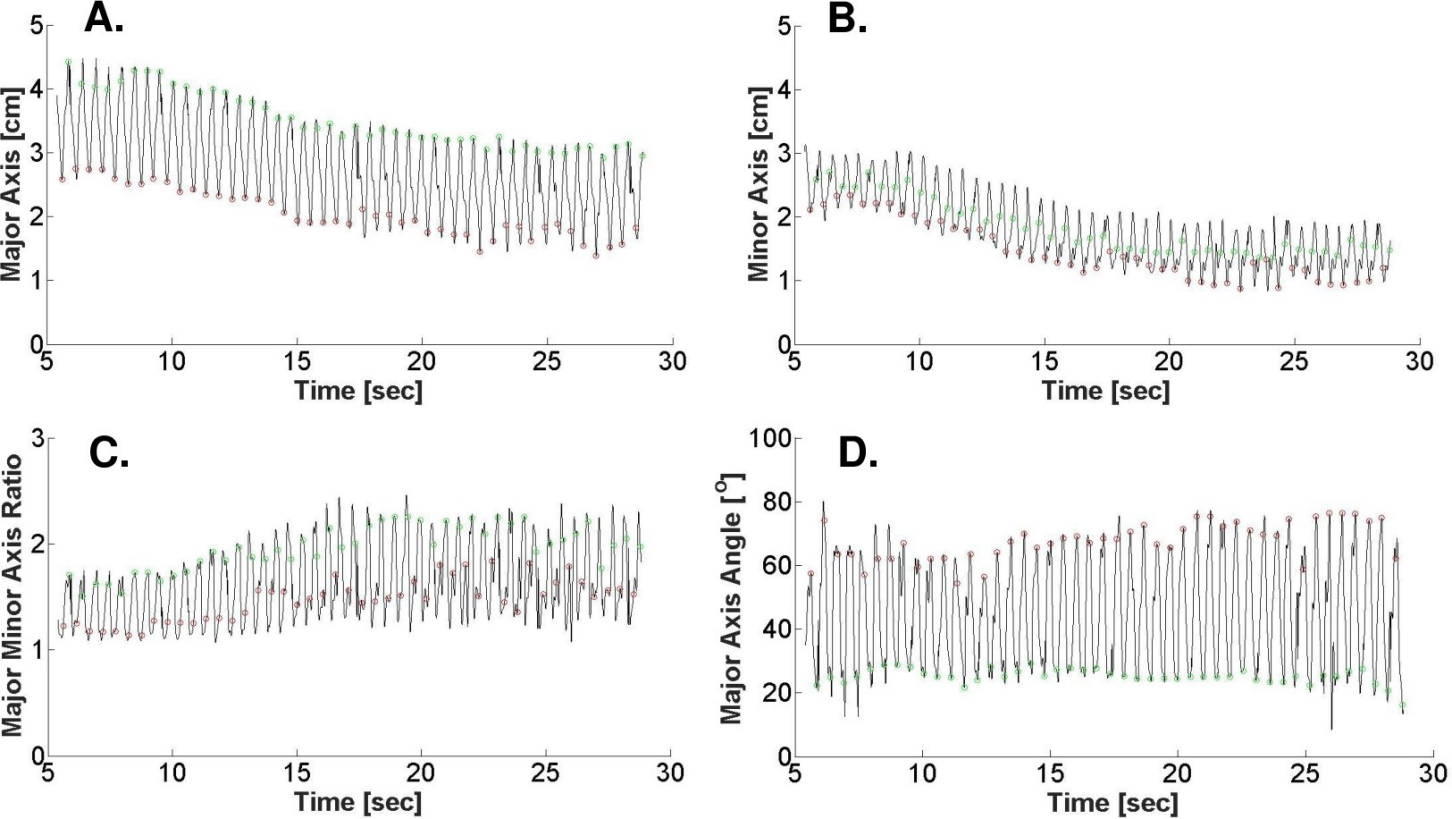
Changes in LV geometry before and during VCO. Shape changes were quantified by 4 indicators calculated from the fitted ellipse: **(A)** Major Axis length, **(B)** Minor Axis length, **(C)** Major to Minor Axis Ratio, and **(D)** Major Axis Orientation Angle. Green makers = end-diastole (ED), Red markers = end-systole (ES).

The change in LV cross-sectional area, major and minor axes, axis ratio, and major axis orientation during VCO are shown in **Table 2**. Also, **Fig 5** shows representative changes in major axis, minor axis, axis ratio, and major axis orientation angle at mid-ventricle LV level during VCO. Briefly, all 5 parameters changed significantly during VCO. The significant change in axis ratio documents that the LV becomes more elliptical during VCO and the significant change in major axis orientation shows that the major axis shifts during VCO but remains generally oriented in the septo-lateral direction.

There were statistically significant differences between LV levels in the rate of change of LV area (-0.08+0.02 cm^2^/ beat apex vs -0.21+0.04 cm^2^/ beat base level) and major axis orientation (-1.04+0.46 ^o^/ beat apex vs 0.81+0.27 ^o^ / beat base) during VCO. There was no difference in major and minor axis or axis ratio between LV levels during VCO.

**Fig 6:**
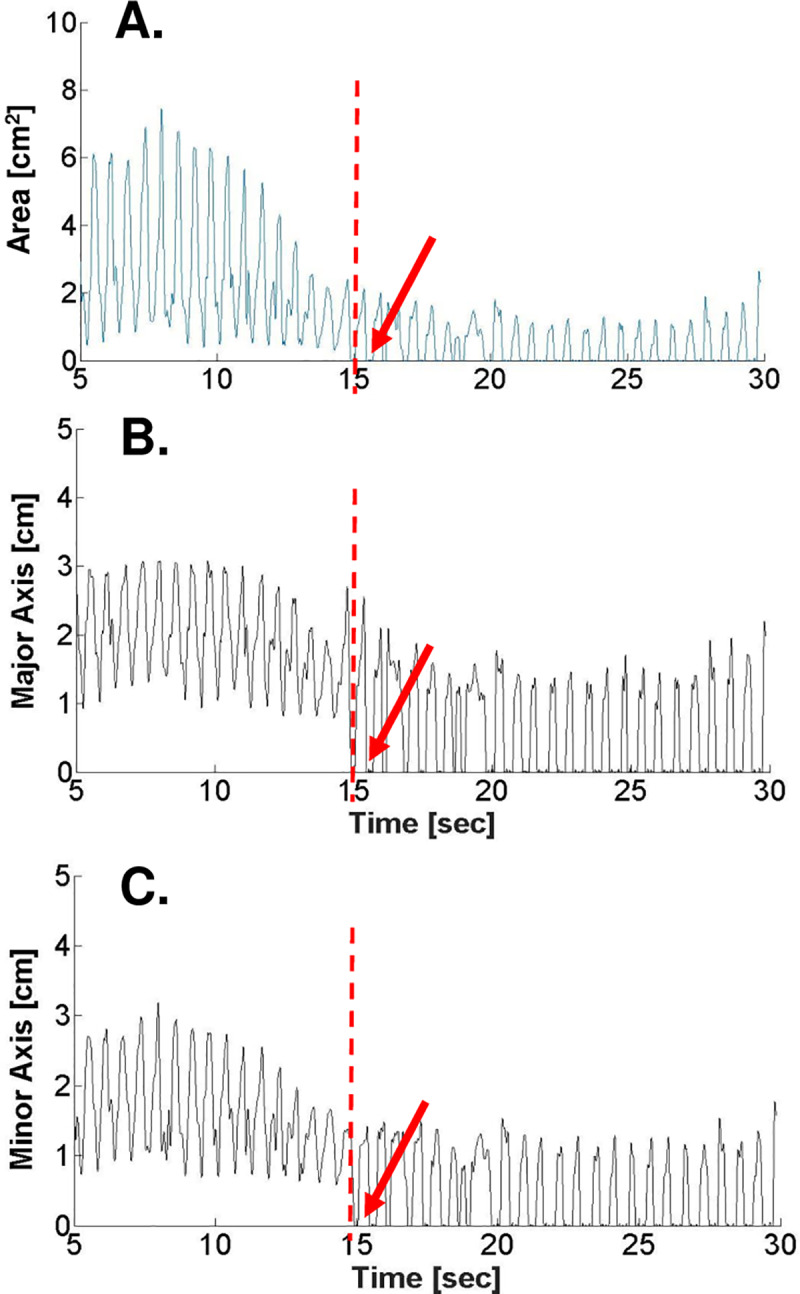
Plots of LV apical area **(A)**, Major axis length **(B)**, and Minor axis length **(C)** of a case showing segmental collapse where LVA < 0.25 cm^2^ after time = 15 sec as indicated by a red arrow.

Last, LV collapse at the apical segment during VCO was observed in 7 of the 10 cases (27 beats). **Fig 6** shows representative plots of LV area, major axis, and minor axis at the apical level. In each case, the dashed red line represents the time when LV cross sectional area fell below 0.25 cm^2^.

### ESPAR

**Fig 7:**
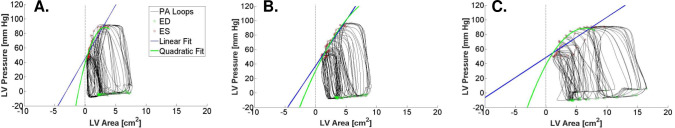
Representative LVP-area (LVPA) loops derived from the level set algorithm and pressure measurements along with fitted ESPAR at apex **(A)**, mid-ventricle **(B)**, and base **(C)** during VCO. The ESPAR was fitted with a linear function (blue) and a quadratic function (green) for comparison. The vertical dashed line represents collapse of the LV to zero volume.

**Fig 7** shows representative LV pressure area loops during VCO at the apex, mid-ventricle and base levels. PA loops shift to the left and the slope of the ESPAR becomes steeper as the LV level moves from base to apex.

**Fig 8:**
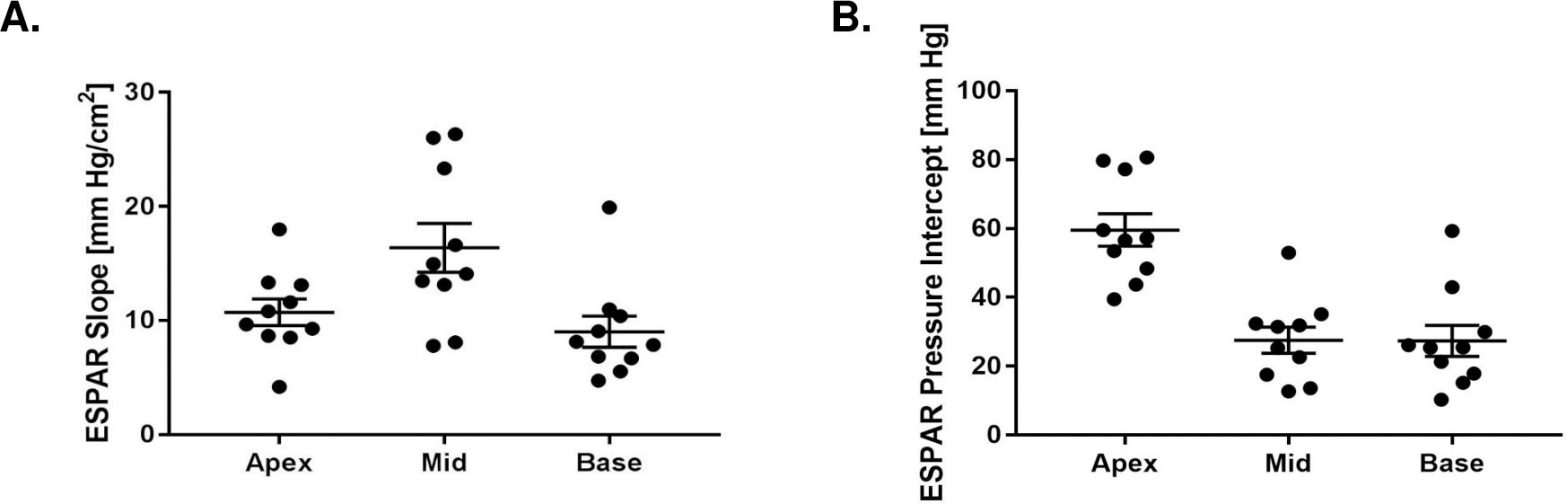
Slope of ESPAR **(A)** and LV area intercept A_o_ **(B)** at apex, mid, and base LV levels for all animals indexed to body surface area.

ESPAR slope and LV pressure intercept are illustrated in **Fig 8A**, **Fig 8B**, and **S1 Table**. The LV pressure intercept for the apex level was significantly higher than mid-ventricle (12.02 mm Hg) and base (8.39 mm Hg) levels mirroring the fact that the apical cavity obliterates before mid and base levels. The mixed-effect model showed that the ESPAR was non-linear (β_2_ = -0.500 (p<0.001), β_1_ = 9.209 (p<0.001), β_0_ = 48.73 (p<0.001)).

### ESPVR

**Fig 9:**
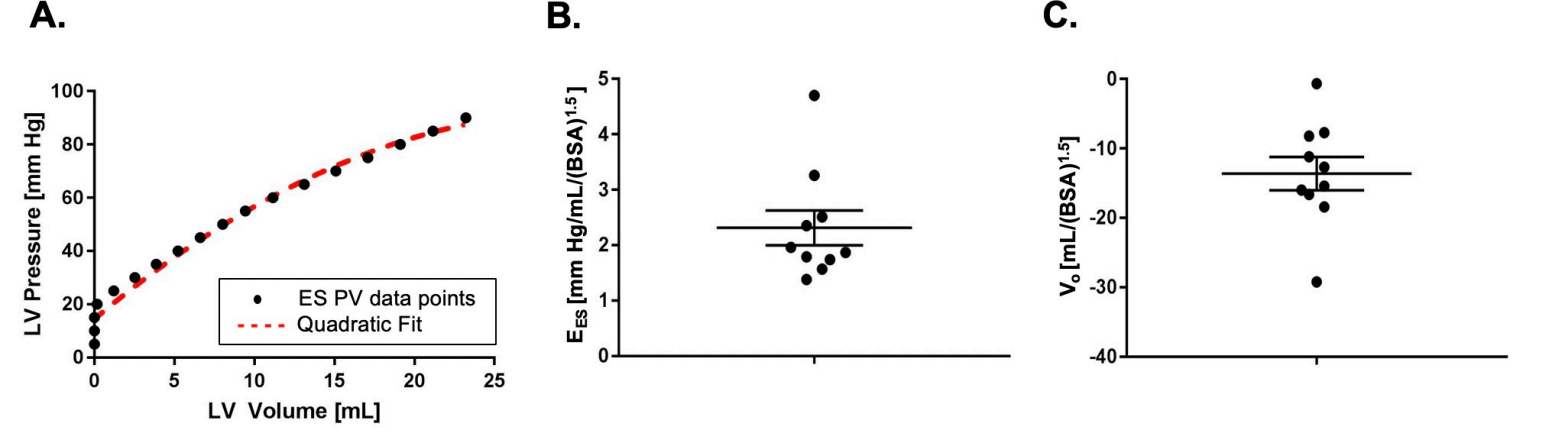
Representative ESPVR with a quadratic fit in red dashed line **(A)**. End-systolic elastance E_ES,Index_ **(B)** and volume intercept V_o,Index_ **(C)** of ESPVR indexed to body surface area.

ESPVR, calculated using a trapezoidal rule method (**Eq 1**), are shown in **Fig 9** and **S2 Table**. A representative ESPVR is shown in **Fig 9A**. Overall, we found E_ES_ to be extremely reproducible between animals (E_ES,Index_ = 2.31± 0.31 mm Hg/ml/m^2^ **(Fig 9B)**; V_o,Index_ = -13.6±2.41 mL/m^2^ **(Fig 9C)**). The mixed-effect model showed that the ESPVR is nonlinear (β_2_ = 0.0414 (p<0.001), β_1_ = 0.349 (p<0.001), β_0_ = 18.12 (p<0.001)).

Real time-based LV volume at ES was compared with the LV volume at ES from the corresponding cine MRI sequence and there was a small but significant offset (35.3±3.6 ml RT vs 43.1±2.4 ml cine p = 0.0047).

### Comparison with conductance catheter

**Fig 10:**
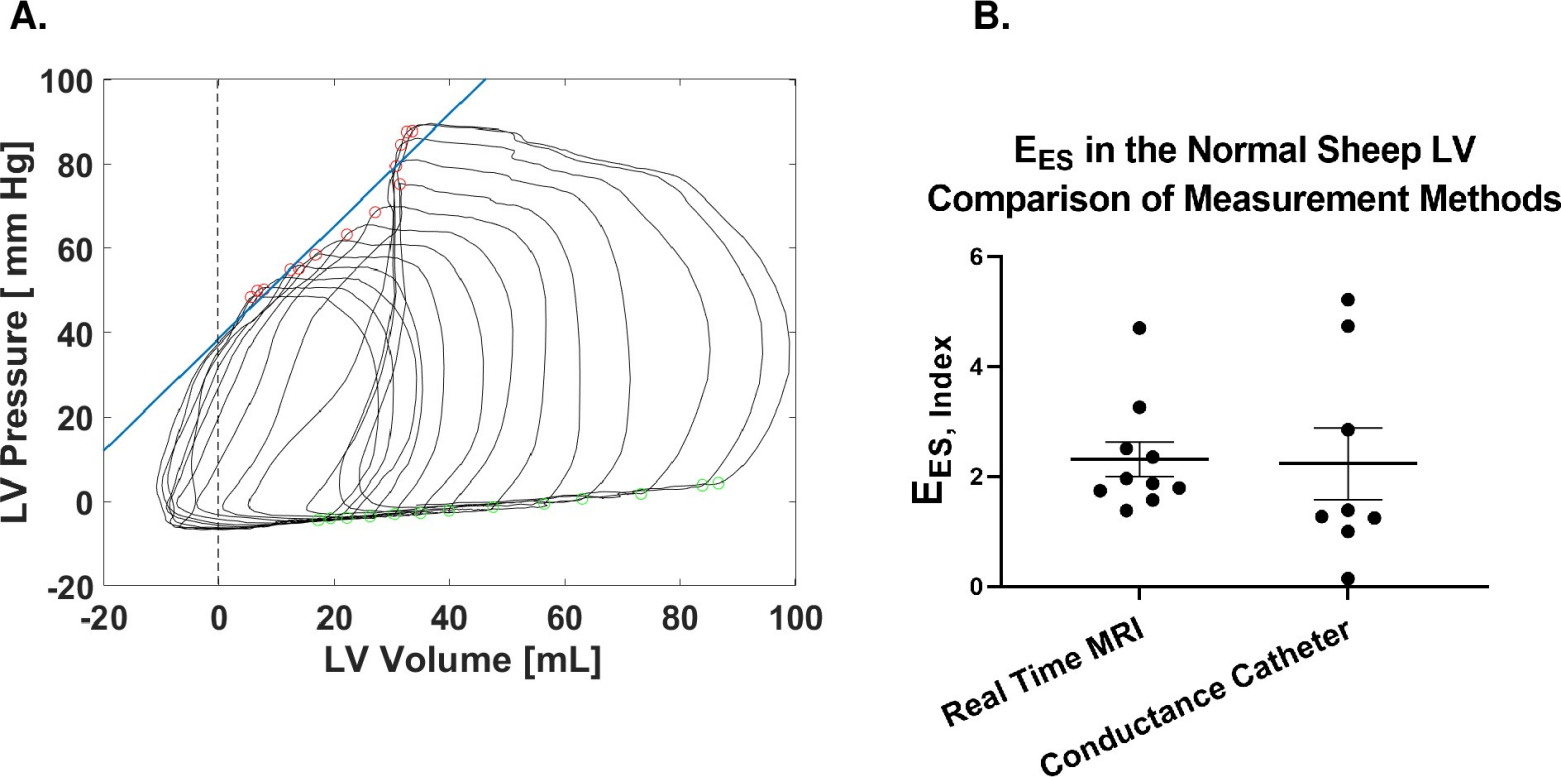
Representative conductance catheter PV loops **(A)**. Comparison of E_ES,Index_ measured with RT and conductance catheter methods **(B)** indexed to body surface area. Green makers = end-diastole (ED), Red markers = end-systole (ES).

Representative LV pressure volume loops during VCO determined with a conductance catheter are shown in **Fig 10A**. As seen in **Fig 10B**, conductance catheter-based measurement of E_ES_ indexed to body surface area and was not significantly different than real time (E_ES,Index_ = 2.31± 0.31 mm Hg/ml/m^2^ by RT vs E_ES,Index_ = 2.23± 0.66 mm Hg/ml/m^2^ by conductance catheter) although the variance of the conductance measurements was higher.

## Discussion

The principal finding of the study is that the RTPVR method allows measurement of LV geometry during VCO and calculation of corresponding ESPAR and ESPVR in normal sheep. Specific findings included that the LV shape becomes more elliptical during VCO leading to eventual apical LV cavity obliteration and that ESPAR and ESPVR are non-linear.

### Semi-automatic active contouring

In the current study, an LSSPM software tool proposed by Wang et al. [19] was used to automatically segment the LV during VCO. As above, the LSSPM has been validated with an MRXCAT phantom [25] and 17 human MRI studies with over 5000 2D images and achieved the highest Dice value [26] compared with three state-of-the-art published methods. In this study, LSSPM was validated by manual segmentation performed in a blinded manner by 2 readers who contoured 200 slices from all 10 subjects (20 slices for each case) and Dice agreement between LSSPM and manual contouring was excellent (87.31%±2.51% and 88.13%±3.43% respectively for the 2 readers).

### LV geometry and apical level cavity obliteration during VCO

The effect of VCO on LV shape during ESPVR measurement has not been previously studied. However, similar to our findings, existing LV geometry data changes in a non-concentric fashion during VCO. For instance, Olsen et al. showed that the anterior posterior (AP) minor axis decreases more than the septal-lateral (SL) minor axis during VCO [36]. Furthermore, LV cavity obliteration occurs in the setting of intra-operative hypovolemia [37] and it therefore seemed likely that LV cavity obliteration occurs during VCO.

The reason for the increased LV ellipticity during VCO where the major axis orientation is in the septo-lateral direction is unclear but could be due to either differential structural stiffness or transmural pressure in the interventricular septum. We did not measure right ventricular (RV) pressure. However, Olsen et al measured LV and RV pressure during VCO in conscious normal dogs and found that the ratio of LV to transmural septal pressure at ES is relatively constant (Calculated from Olsen Fig 5) [38]. Further work is warranted but this finding suggests that the septum may be structurally different than the LV free wall.

### Comparison with other RTMRI-based studies of ESPAR

Contijoch et al. observed an increase in ESPAR pressure intercept at the LV apex in their RTMRI based study in normal sheep but the slope of ESPAR relationship in that study was not different between LV levels [18]. In contrast, we found statistically significant variations in ESPAR slope, curvature and intercept between LV levels (**Fig 8**) as well as cavity obliteration at the apex. The reason for the difference is unclear. We did use a neosynephrine infusion to counteract anesthesia related hypotension and maintain peak LV pressure at 90 mm Hg and as a consequence, LV pressure change during VCO in our study was somewhat higher.

### ESPVR

#### Comparison of RTMRI and conductance catheter-based measurement of E_ES_

We found no significant difference between RTMRI and conductance catheter measurements of E_ES_ although there was greater variance in the conductance catheter measurements. We recognize that the study did not have 3D RTMRI as a gold standard for comparison. However, the decreased variance suggests that RTMRI is a more accurate measure of E_ES_ than conductance catheter-based measurement.

#### ESPAR and ESPVR non-linearity

It is now generally accepted that the ESPVR is non-linear. For instance, Kass et al used sonomicrometry [11] and van der Velde et al used conductance catheter methods [39] to measure ESPVR in open chest dogs and found that ESPVR is convex with the center of curvature to the right and with a quadratic term (β_2_) between -0.041 (Calculated for control values from van der Velde Table 2) [39] and -2.68 mm Hg/ ml^2^ [11]. We found that both ESPAR at all LV levels and ESPVR were significantly nonlinear with ESPVR curvature in our study (β_2_ = -0.0414) identical to van der Velde but significantly less than values obtained by Kass. Further study is warranted but it is our prejudice is that the β_2_ non-linearity term in our study is likely to be more accurate because of the greater spatial accuracy of the RTMRI method.

#### Relationship between ESPVR and systolic myocardial stiffness

Implicit in ESPVR analysis is the assumption that there is a relationship between the pressure volume relationship and systolic myocardial stiffness [1]. Equations for systolic myocardial stiffness have been previously derived [40]. However, we contend that a more valuable approach would be to use the entire ESPVR as input into an inverse finite element (FE) model-based optimization of regional systolic myocardial stiffness [41]. FE simulations of end systole are currently optimized with only the end-systolic pressure volume point and do not take advantage of the rich amount of data contained in the ESPVR.

**Fig 11:**
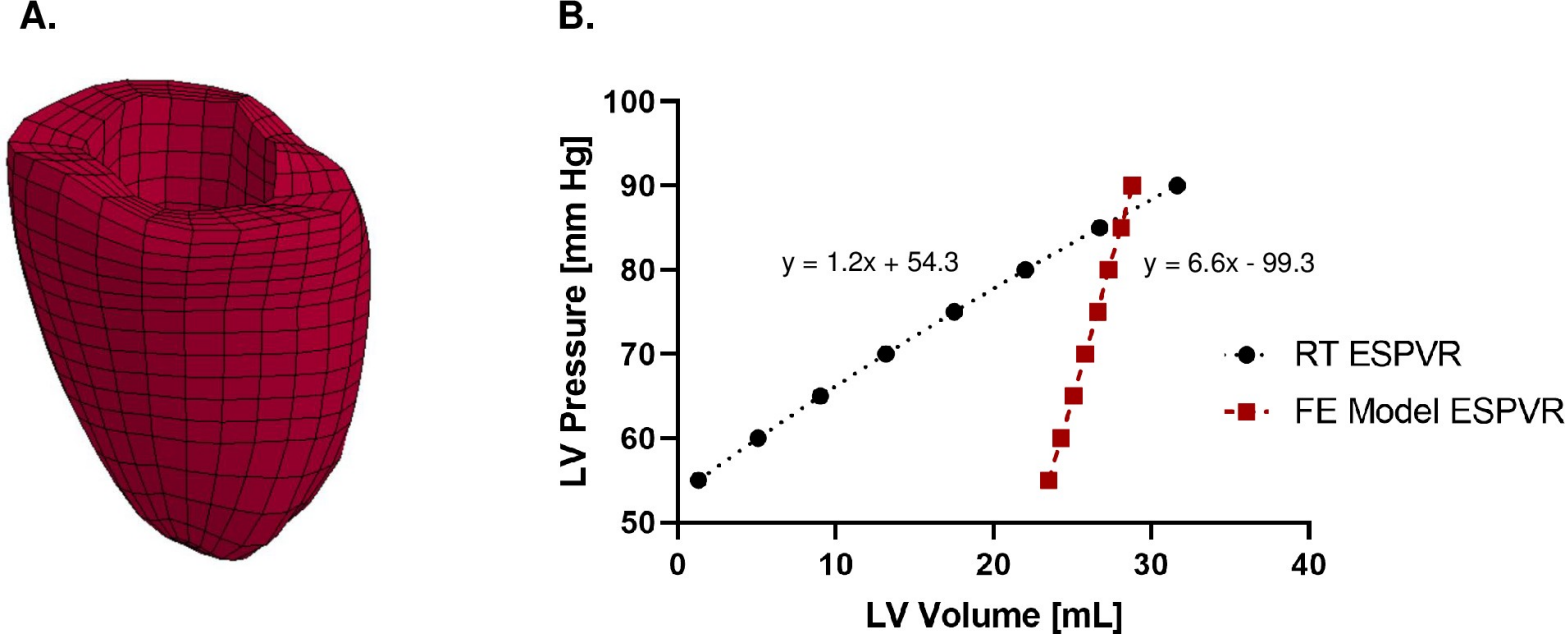
FE mesh of a representative sheep case **(A)**. Comparison between FE based calculation of ESPVR and experimental RT ESPVR **(B)**.

As a case in point, we present in **Fig 11** a comparison between ESPVR measured using RT and that predicted using a sheep-specific FE model where the RT and FE model are based on the same animal. Briefly, the FE model was created using established methods [42] where a mesh of hexahedral elements was created from the animal-specific CineMRI **(Fig 11A)**. Diastolic filling and systolic contraction were simulated using the passive and active material laws previously described by Guccione et al [43, 44]. Global myocardial tissue stiffness and contractility were further optimized to best match end-diastolic and end-systolic volume measured from cine MRI prior to VCO. To simulate ESPVR, a series of systolic contraction simulations were run by varying LV pressure at ES from 50 mmHg to 90mmHg and recording the simulated end-systolic LV volume. As **Fig 11B** illustrates, the FE model predicted ESPVR exhibits a significantly steeper E_ES_ (slope = 6.6) compared to the corresponding RT measured E_ES_ (slope = 1.2). Briefly, this well-established Guccione active contraction law-based [43] model is accurate at the ES pressure and volume at which it was optimized but fails to match the ESPVR at lower ES pressures. The Guccione active contraction material law [43] has a number of parameters and future work will include individual optimization of those parameters and/ or revision of the contraction law proper. However, this example reinforces the utility of our geometrically accurate RT ESPVR method to inform the optimization and design of myocardial active contraction material laws and hence to improve the simulation of cardiac systole.

#### RT measurement of the ED pressure volume relationship (EDPVR)

Although the focus of the current study is the ESPVR, measurement of the end-diastolic pressure volume relationship (EDPVR) is equally important [45]. First, the EDPVR together with ESPVR determines LV pump function via the Frank-Starling mechanism [46]. In addition, diastolic LV dysfunction is common, increasing with age [47] to reach 36% in patients that are 76 years old [48]. Furthermore, diastolic dysfunction increases the risk of heart failure [47, 48] where the incidence of diastolic heart failure may be increasing over time [49].

Similar to ESPVR, seminal studies of EDPVR used sonomicrometry [50] and conductance catheters [51]. However, RTMRI has recently been used to measure EDPAR [18] and additional RTMRI-based studies of EDPVR and LV shape change during altered LV loading are warranted. For instance and similar to the finite element optimization using RT ESPVR above, RTMRI-based EDPVR could be used to optimize finite element calculations of myocardial diastolic material parameters furthering our understanding of global and regional diastolic function, diastolic dysfunction and diastolic heart failure.

### Limitations

With the current method we are able to acquire 10.76 images per beat which are then interpolated to create PA loops. Further, synchronization between pressure and area is based on pressure and area metrics rather than the ECG. **S1 Fig** addresses this issue by showing that shifting of the pressure and area alignment did not affect the ESPAR calculation. We did note a small but significant offset in LV volume at ES when RT-based measurements were compared to the corresponding cine MRI sequence. We believe this offset is due to our use of a trapezoid rule-based approximation of the LV contour (Eq 1) while the cine MRI images were manually segmented with a spline. Last, LSSPM segmentation of the LV long axis during VCO was thought to not be possible because of algorithm failure to distinguish LV and left atrium at the mitral valve level.

## Conclusions and future directions

In conclusion, LSSPM segmentation of 2D RT MRI images is accurate and allows measurement of LV geometry during VCO and calculation of corresponding ESPAR and ESPVR. Although the goal was to describe the RT ESPVR method, it was noted that LV shape becomes more elliptical during VCO leading to eventual apical LV cavity obliteration and that ESPAR and ESPVR are non-linear.

Future work will focus on semi-automatic segmentation of the LV long axis during VCO with machine learning based methods. Hopefully, more rapid image acquisition as well as actual 3D real time methods will also be developed. Next, RTESPVR will be used to determine the accuracy of single beat ESPVR methods. Last, similar RTPVR methods should be developed for the EDPVR relationship and, as before, the RTPVR will facilitate determination of the relationship between ESPVR and underlying regional systolic material parameters.

## Supporting information

S1 Fig Alignment using max + dLVP/dt and max LV area was initially chosen because of the perception that beat shape was more appropriate. In retrospect, there is little effect between this (A) and 0.25 max + dLVP/dt and max LV area (B).
(TIF)

S1 Table Slope and volume intercept of ESPAR at three LV levels derived from RTMRI-based analysis. (DOCX)

S2 Table End-systolic elastance E_ES_ (slope) and volume-axis intercept (V_o_) estimated from the ESPVR indexed to body surface area (BSA)^1.5^. (DOCX)

S1 Data (XLSX)
