# Supplementary figures and images for: Left ventricular geometry during unloading and the end-systolic pressure volume relationship: Measurement with a modified real-time MRI-based method in normal sheep
Source: PLoS One. 2020 Jun 22;15(6):e0234896. doi: 10.1371/journal.pone.0234896 (PMC7307770; doi:10.1371/journal.pone.0234896)

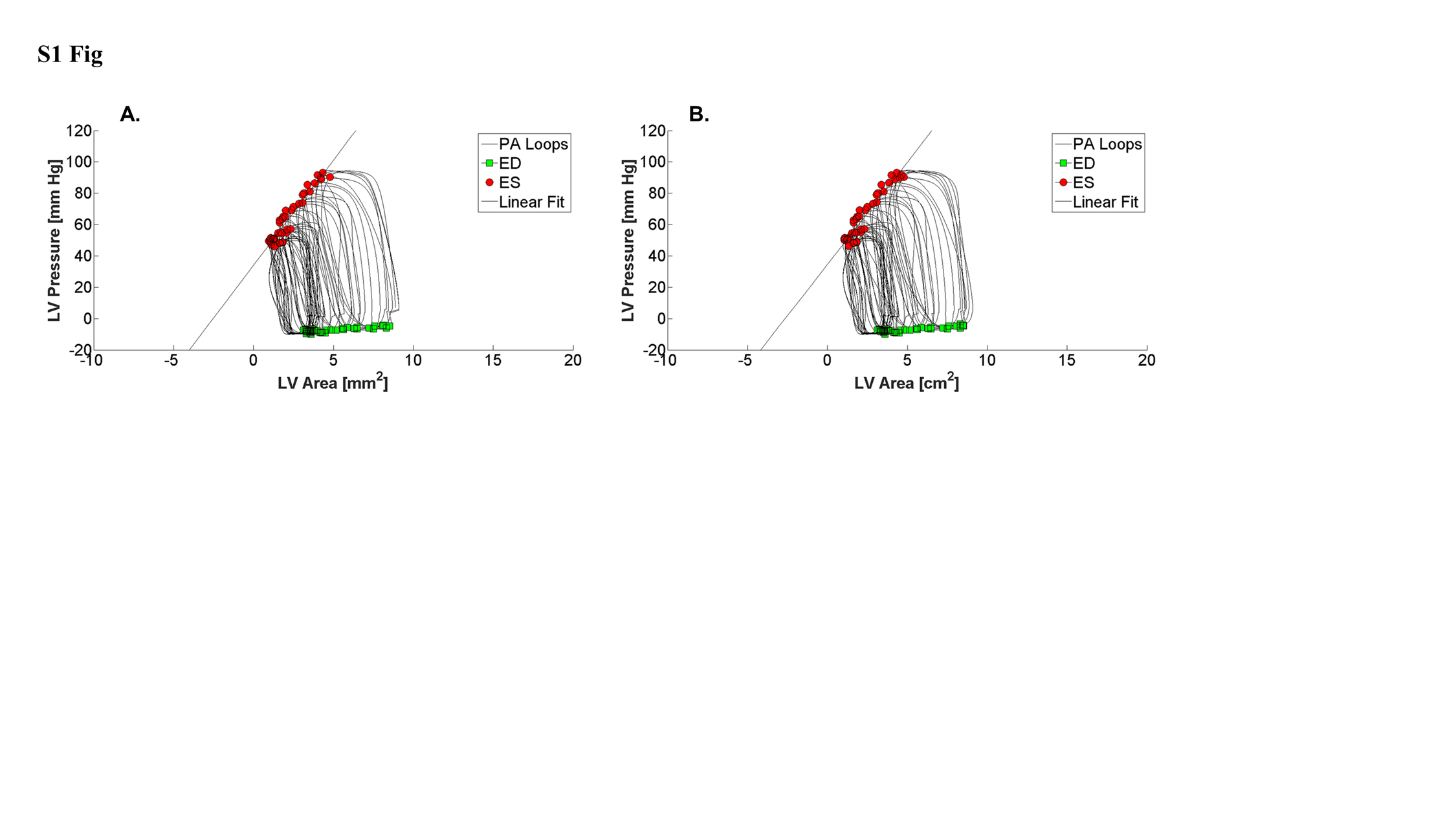

Supplement: S1 Fig — In retrospect, there is little effect between this (A) and 0.25 max + dLVP/dt and max LV area (B). (TIF) [file pone.0234896.s001.tif]
