# Supplementary material for: Left ventricular geometry during unloading and the end-systolic pressure volume relationship: Measurement with a modified real-time MRI-based method in normal sheep
Source: PLoS One. 2020 Jun 22;15(6):e0234896. doi: 10.1371/journal.pone.0234896 (PMC7307770; doi:10.1371/journal.pone.0234896)
Supplement: S1 Table — (DOCX) [file pone.0234896.s002.docx]

## S1 Table. Slope and volume intercept of ESPAR

|  | **ESPAR Slope [mm Hg/cm^2^]** | | | **A_o_ [cm^2^]** | | |
| --- | --- | --- | --- | --- | --- | --- |
| **Animal #** | **Apex** | **Mid** | **Base** | **Apex** | **Mid** | **Base** |
| 1 | 4.18 | 26.3 | 11.0 | -18.5 | -0.858 | -1.63 |
| 2 | 11.6 | 23.4 | 9.07 | -4.87 | -0.543 | -2.80 |
| 3 | 9.67 | 8.08 | 6.68 | -4.52 | -0.395 | -2.28 |
| 4 | 18.0 | 16.6 | 8.13 | -2.97 | -0.820 | -3.69 |
| 5 | 13.3 | 13.5 | 4.75 | -3.63 | -2.61 | -12.5 |
| 6 | 9.28 | 7.80 | 6.84 | -4.25 | -4.04 | -1.50 |
| 7 | 10.8 | 26.0 | 19.9 | -5.29 | -0.675 | -1.07 |
| 8 | 8.67 | 13.1 | 7.86 | -9.20 | -4.03 | -3.32 |
| 9 | 13.1 | 15.0 | 5.54 | -4.55 | -1.69 | -7.75 |
| 10 | 8.51 | 14.1 | 10.4 | -9.48 | -2.30 | -2.46 |
| Mean + SEM | 10.7 + 1.16 | 16.4 + 2.13 | 9.02 + 1.36 | -6.73 + 1.48 | -2.15 + 4.59 | -3.90 + 1.12 |
