# Supplementary material for: Left ventricular geometry during unloading and the end-systolic pressure volume relationship: Measurement with a modified real-time MRI-based method in normal sheep
Source: PLoS One. 2020 Jun 22;15(6):e0234896. doi: 10.1371/journal.pone.0234896 (PMC7307770; doi:10.1371/journal.pone.0234896)
Supplement: S2 Table — (DOCX) [file pone.0234896.s003.docx]

## S2 Table. End-systolic elastance E_ES_ and volume-axis intercept

| **Animal #** | **E_ES,Index_ [mm Hg/mL/m^2^]** | **V_o,Index_ [mL/m^2^]** |
| --- | --- | --- |
| 1 | 3.26 | -8.21 |
| 2 | 1.87 | -16.0 |
| 3 | 1.38 | -18.4 |
| 4 | 2.35 | -12.7 |
| 5 | 1.74 | -29.2 |
| 6 | 1.57 | -15.4 |
| 7 | 2.51 | -11.2 |
| 8 | 1.96 | -16.6 |
| 9 | 1.79 | -6.64 |
| 10 | 4.70 | -7.73 |
| Mean + SEM | 2.31+0.31 | -13.6+2.41 |
